# Ultra-broadband and strongly enhanced diffraction with metasurfaces

**DOI:** 10.1038/srep10119

**Published:** 2015-05-14

**Authors:** Yong Zhang, Lin Zhou, Jia-qi Li, Qian-jin Wang, Cheng-ping Huang

**Affiliations:** 1 Department of Applied Physics, Nanjing Tech University, Nanjing 210009, P.R. China; 2 National Laboratory of Solid State Microstructures, Nanjing University, Nanjing 210093, P. R. China; 3 Department of Physics, Southeast University, Nanjing 211189, P. R. China

## Abstract

Enhanced high-order diffractions which are spatially dispersive are desirable in such as spectroscopy studies, thin-film solar cells, etc. Conventionally, the dielectric gratings can be used to realize the enhanced diffraction, but the facets are usually rugged and optically thick (~μm). Plasmonic materials may exhibit unprecedented ability for manipulating light. Nonetheless, much interest has been focused on the subwavelength metasurfaces working in the zero-order regime. Here, we show that ultra-broadband and strongly enhanced diffraction can be achieved with the super-wavelength metasurfaces. For the purpose, we employ symmetric or asymmetric metal patches on a ground metal plane, which support the localized oscillation of free electrons and enhanced scattering of light. The zero-order reflection is suppressed, giving rise to an enhancement of first-order diffraction (50 ~ 95%) in an ultra-wide bandwidth (600 ~ 1500 nm). The proposed plasmonic structure is planar and ultra-thin (with an etching depth of only 80 nm), showing new potential for constructing compact and efficient dispersive elements.

Metal nanostructures provide great potential for manipulating light, because of the strong coupling between electromagnetic fields and surface charges. As building blocks of metasurface and metamaterial designs, isolated nanoholes in a metal or nano metal-particles in a dielectric can induce surface-plasmon polariton (SPP) mode or localized surface-plasmon (LSP) resonance^2^. With periodic array of nanoholes or nanoparticles, enhanced optical transmission, polarization conversion, negative or high index, and anomalous refraction etc. have been suggested^9^. The metal nanostructures such as metal-insulator-metal sandwiches can also be used to construct the electromagnetic wave absorbers^4^, which may find a host of potential applications in solar cells, photodetectors, bolometers, etc. Up to date, most of research interests have been focused on subwavelength plasmonic system, where the lattice period is smaller or much smaller than the working wavelength^9^. In the metamaterial studies, this enables the construction of an effective medium with exotic optical properties. For metasurfaces composed of small apertures, the subwavelength features will suppress the high-order modes, giving rise to enhanced zero-order diffraction in certain wavelength regions^3^.

In some particular cases such as the spectroscopy study and applications, enhanced (propagating) high-order diffractions which are angularly dispersive are desirable. In thin-film solar cells, microstructures with enhanced diffraction can also play the role of anti-reflecting coatings and convert the incident light via diffraction into the waveguide modes of dielectric spacer^0^. Conventionally, the dielectric diffraction gratings with triangle profiles or deep rectangular grooves may exhibit enhanced diffraction in certain conditions^4^. However, as the facets are rugged and optically thick, the fabrication of gratings becomes difficult. Recently, researchers started to manipulate the diffraction by using the plasmonic structures. For example, with wavelength-scale stepping on the surface of a bulk negative-index metamaterial, enhanced first-order diffraction has been demonstrated by Smith *et al.*^5^. More recently, Guo *et al.* investigated a dual-period plasmonic grating, where a larger-period modulation was imposed on the small-period slit or hole arrays in a metal film^7^. The system can support the propagating first-order diffraction near the SPP resonance. Nonetheless, the reported working bandwidth is very narrow (~100 *nm*) and the diffraction efficiency is small (less than 5%). Indeed, the increase of lattice period will not necessarily lead to the efficient diffraction.

Here, we show that ultra-broadband and strongly enhanced diffraction of light can be realized with the periodic quasi-two-dimensional plasmonic nanostructures. The lattice period in one direction of the metasurface is subwavelength and in the other direction is super-wavelength (here “super-wavelength” means that the period is larger than the light wavelength). We found that, with the unit cells of symmetric or asymmetric metal-dielectric-metal sandwiches, wideband and enhanced scattering of light can be induced. Consequently, the first-order diffraction is greatly boosted (with an efficiency of 50 ~ 95%) in an ultra-wide frequency band (600 ~ 1500 *nm*), due to the interference effect of periodic super-wavelength structures. The proposed plasmonic structure is planar, ultra-thin (the etching depth is only 80 *nm*), and it works at the normal incidence. This facilitates the fabrication of structure and operation of the effect. The unique characters of the structure become attractive for constructing new and efficient devices for dispersing light.

## Results

### Rectangle metal patch array

**Figure 1:**
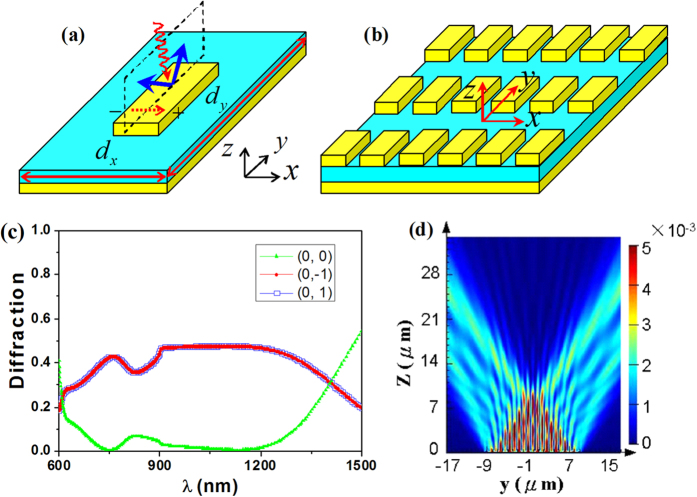
(**a**) Three dimensional illustration of a unit cell of metal sandwiches. (**b**) Top view of metal sandwiches with the rectangle patch arrays. (**c**) Simulated diffraction spectra of the structure. (**d**) Simulated energy-flow distribution at the wavelength 1050 nm.

In the metal-dielectric-metal sandwiches, the top periodic metal patches and the bottom planar metal screen are separated by a thin glass spacer. Here, the top metal layer is set as the rectangle metal patches. Fig. 1(a) present the schematic illustrations of unit cell and the patches array, respectively. The length, width, and thickness of the rectangle patch were chosen, respectively, as *l* = 600 *nm*, *w *= 200 *nm*, and *h* = 80 *nm*. The thickness of the glass spacer is *t* = 90 *nm* (the qualitative conclusion does not depend on the sizes of unit cell; see the supporting information). The bottom metal layer is 150 *nm* thick, which is much larger than the skin depth and can prevent the light transmission. The frequency band we are interested in ranges from the visible to infrared regions with λ ~ 600–1500 *nm*. The lattice period in the *xy* plane were set as *d*_x_ = 360 *nm* and *d*_y_ = 1800 *nm*, which is subwavelength in *x* direction and super-wavelength in *y* direction. The plane wave is incident normally (from the top side) upon the structure with the electric field along the *x* axis. The reflection spectra of system were simulated with the finite-difference time-domain (FDTD) method.

Figure 1(c) shows the simulated spectra of diffraction of the metasurface. Here, only the zero-order (0, 0) mode and first-order (0, 1) and (0,−1) modes are shown in the spectra; the higher-order diffraction modes are found to be very weak. The two first-order diffractions are degenerate due to the symmetry of the structure. Interestingly, in a broad bandwidth, the zero-order reflection is suppressed while the first-order diffractions are significantly increased. Explicitly, in the spectral range 650 ~ 1300 *nm*, the reflectivity of the zero-order mode is lower than 10%, and the efficiency of the first-order mode attains 47% (the sum of the two first-order modes reaches 94%). At the shorter or longer wavelength (λ < 650 *nm* or λ > 1300 *nm*), however, the first-order diffractions will be less predominant. To observe the effect more visually, Fig. 1(d) presents the simulated energy-flow distribution (in yz plane) for the wavelength λ = 1050 *nm*. One can see that two symmetrical diffraction orders are produced from the metamaterial surface. The diffraction angle is calculated to be 35.0 degrees.

**Figure 2:**
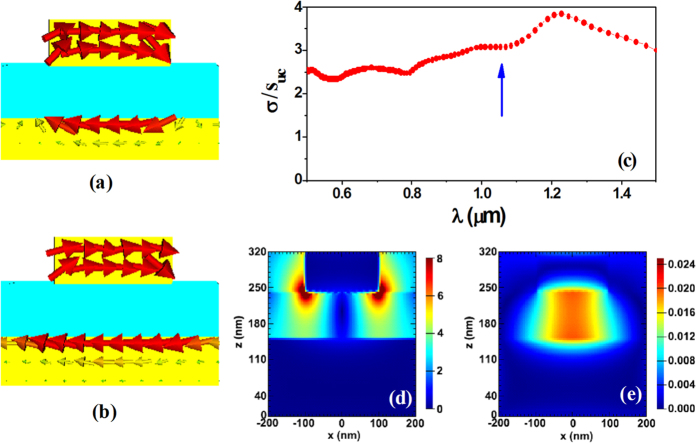
Simulated current distributions for the periodic metal sandwiches with rectangle patches (on the central cross section of unit cells, in xz plane) at the wavelength: (**a**) 830 nm and (**b**) 1030 nm. (**c**) Normalized scattering cross section, (**d**) electric- field and (**e**) magnetic-field patterns of a single unit cell. Here, the near fields in (**d**) and (**e**) correspond to the wavelength 1050 nm (marked in (**c**) by an arrow).

An interesting question is that how can a simple rectangle metal-patch array above a planar metal plate possess ultra-broadband diffraction spectrum. Generally, a periodic structure cannot own a resonance with ultra-wide resonance width. To answer this question, the current distributions of the periodic structure (on the central cross section of unit cells) have been calculated. Here, we take the wavelength 830 *nm* and 1030 *nm* as two examples and the obtained results are shown in Fig. 2(a), respectively. We can see that in both cases anti-parallel currents are induced in the upper metal patch and the lower ground metal plane, thus forming a current loop. The wavelength 830 *nm* corresponds to a shorter circumference of current loop while the wavelength 1030 *nm* corresponds to a longer one. We also performed the simulations for other wavelengths and similar results can be seen: A current loop is produced in the metal sandwiches; the larger the excitation wavelength, the longer the circumference of the current loop. This suggests that for any wavelength a localized and self-adaptive oscillation of free electrons can be induced in the unit cells.

To confirm the localized oscillation which is due to unit cell rather than the periodicity, we numerically calculated the scattering spectrum of a single unit cell. Fig. 2(c) presents the calculated scattering cross section (normalized to the area of unit cell) as a function of wavelength. In the considered wavelength region, the normalized scattering cross section is around 2.3 ~ 3.8, indicating a wideband and enhanced scattering of the incident light. Actually, the wideband and enhanced scattering has been seen for some cases. For example, in the Thomson scattering, a free electron can scatter the electromagnetic fields with a normalized scattering cross section (normalized to the classic geometric cross section of electron) of 8/3, independent of the wavelength (at the low frequencies)^8^. Here, the plasmonic materials containing free electrons could play a similar role. Compared with a single metal particle such as the nanosphere or nanorod, the nanosandwich structure can adjust its loop length effectively to respond to the external excitation (see Fig. 2(a)), thus generating a resonance-like or quasi-resonance plasmonic response. Taking an arbitrary wavelength such as λ = 1050 *nm* as an example, Fig. 2(d) plots, respectively, the electric-field and magnetic-field patterns for the single unit cell (the incident electric field is set as unity). The enhanced electromagnetic fields or hot spots in the nanosandwich confirm the localized and resonance-like plasmonic response.

Besides the unit cell which exhibits wideband and enhanced scattering, the structure periodicity also plays an important role. When the incident light illuminates normally upon the metal surface, the electromagnetic fields will be scattered by the unit cells. The interference of these scattering fields gives rise to the discrete diffraction orders. The in-plane wavevector of diffraction modes is governed by the reciprocal lattice vector *G*_mn_:


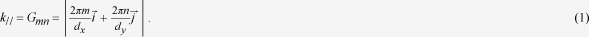


Here, *m* and *n* are two integers. Correspondingly, the diffraction angle θ is determined by the following generalized grating equation:


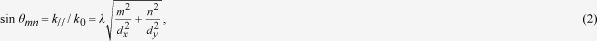


where the real solutions of *θ* correspond to the outgoing or propagating waves. Because of the subwavelength feature in *x* direction (*d*_x_ < λ) and super-wavelength feature in *y* direction (*d*_y_ > λ), only the (0, *n*)-order modes can be propagating and all the other diffraction orders will be evanescent. Thus our design helps to generate a very few propagating modes with the concentrated energy. The (0, *n*)-order modes travel along the yz plane, with the diffraction angle being 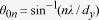 (here, *n* = 0, ±1 denotes, respectively, the zero-order and first-order diffraction). For the first-order mode, the diffraction is angularly dispersive with θ varying from 19.5 to 56.5 degrees. When λ = 1050 *nm*, the equation predicts that the diffraction angle is *θ*_01_ = 35.7 ^o^, close to 35 ^o^ obtained by the simulation (see Fig. 1(d)).

### Trapezoid metal patch array

**Figure 3:**
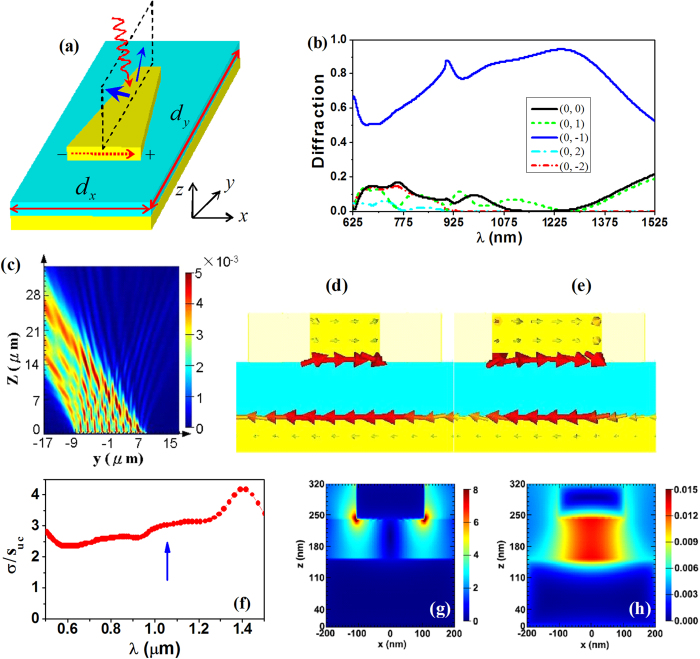
(**a**) Schematic view of unit cell of metal sandwiches with the trapezoid patches. (**b**) Simulated diffraction spectra of the structure. (**c**) Simulated energy-flow distribution at the wavelength 1050 nm. (**d**) and (**e**) The current distributions at different cross sections of the trapezoid patch (at 1050 nm). (**f**) Normalized scattering cross section, (**g**) electric-field and (**h**) magnetic-field patterns of a single unit cell. Here, the near fields in (**g**) and (**h**) correspond to the wavelength 1050 nm (marked in (**f**) by an arrow).

Due to the symmetry of rectangle metal patches, the two first-order diffractions are degenerate, restricting the efficiency of either mode to less than 50%. This is not beneficial to the practical applications. To suppress one of the first-order modes and enhance the other one, an asymmetrical metasurface may be used. We notice that the asymmetric plasmonic structures, e.g., a metal film perforated with subwavelength triangle holes, can be employed to excite the SPP modes asymmetrically on the film surface^9^. Here, a trapezoid patch array on a planar metal plate will be used to realize the asymmetric and enhanced outgoing diffractions. Fig. 3(a) shows the unit cell of the isosceles trapezoid patch array. Compared with the rectangle patch, both unit cell and patch array of the trapezoids are not symmetric with respect to the xz plane any more. In this case, the unit cell will present asymmetrical light scattering in the yz plane. The superposition of scattering waves from the periodic super-wavelength patch array will give rise to the asymmetrical first-order diffractions.

Numerical calculations have been carried out to verify the above idea. In the simulation, the lattice constant and film thickness are the same as those used for the rectangle patches (d_x_ = 360 *nm*, d_y_ = 1800 *nm*, and the thickness of sandwich is 80/90/150 *nm*). The widths of two bases of the trapezoid are chosen, respectively, as 100 *nm* and 320 *nm*, and the length of the trapezoid is selected as 1200 *nm* (the dependence of diffraction spectrum on the unit cell sizes is shown in the supporting information). Fig. 3(b) shows the simulated diffraction spectra of the trapezoid patch array. In the whole spectral range around 625 ~ 1525 *nm*, the zero-order spectrum is very weak with the reflectivity less than 20%. Simultaneously, the two first-order diffractions split due to the asymmetry of the structure. The efficiency of the (0, 1) mode is also lower than 20% in the studied spectral range. In contrast, the (0,−1) diffraction mode is greatly boosted in a wide frequency band: the diffraction efficiency is up to 50-95% and the working frequency expands from 625 *nm* to 1525 *nm* with a bandwidth of 900 *nm*. From 980 *nm* to 1370 *nm*, the efficiency is even higher than 80%. Fig. 3(c) presents the simulated energy-flow distribution for the wavelength 1050 *nm*. The asymmetrical and enhanced first-order diffraction can be seen clearly. In addition, we notice that the second-order diffractions also present in the spectra. Nonetheless, they correspond to the shorter wavelength (625 ~ 900 *nm*), lower efficiency (less than 15%), and the larger diffraction angle (44 ~ 90 degrees).

The analysis given for the rectangle patches can be applied to the trapezoid patches as well. Besides the asymmetry in the structure and scattering, the trapezoid patches can also support a wideband and enhanced scattering of the light. Fig. 3(d) plot the current distributions on different cross sections of unit cells (for the periodic structure; λ = 1050 *nm*). The results suggest that localized oscillations of free electrons can be induced simultaneously at different cross sections of the trapezoid patches. The oscillation of electrons gives rise to the scattering far fields. The normalized scattering cross section and electromagnetic-field distributions (λ = 1050 *nm*) for a single unit cell are also calculated and shown in Fig. 3(f)-(h). The wideband far-field scattering and enhanced near fields of the single unit cell are obvious. The results provide extra degree of freedom for manipulating the diffraction.

**Figure 4:**
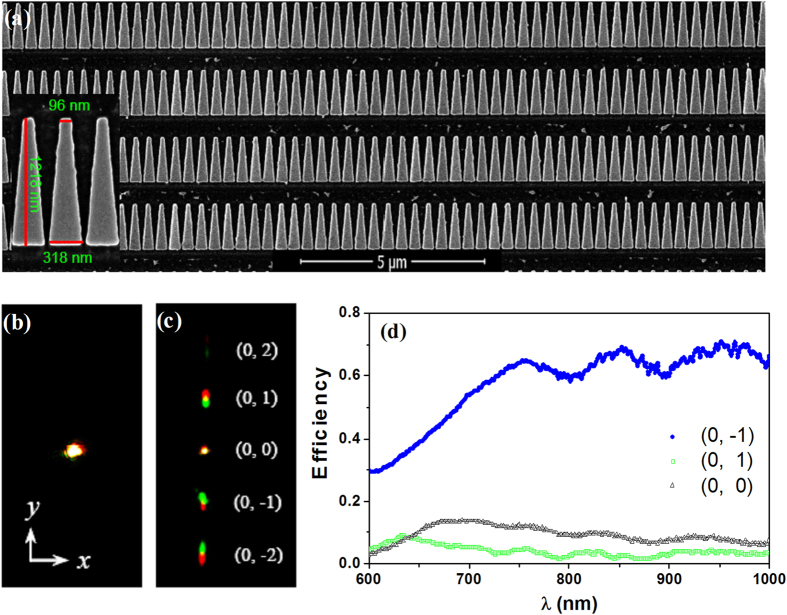
(**a**) FIB images of the trapezoid patch array. (**b, c**) The diffracted optical fields captured by CCD without (**b**) and with (**c**) the trapezoid patches. (**d**) Experimental diffraction spectra of the zero- and first-order modes.

As an experimental demonstration of the above effect, the plasmonic metasurface with the trapezoid patch array has been fabricated with the focused-ion-beam (FIB) system. In the experiment, the structural parameters of the sample were set according to those of the simulated one. Fig. 4(a) shows the part scanning electron microscope (SEM) image of the trapezoid metal patches. Fig. 4(b) present the CCD-captured optical fields reflected or diffracted from a flat metal surface and the plasmonic metasurface, respectively. With the flat metal surface, the incident light is mirror reflected with a well-defined light spot (see Fig. 4(b)). However, when the light is illuminated on the metasurface, the incident light is strongly diffracted which spreads in the space (in y direction or yz plane; see Fig. 4(c)). Here, five diffracted light spots can be seen, corresponding to (0, 2), (0, 1), (0, 0), (0,−1), and (0,−2) diffraction orders (the (0, 2) mode is very weak). Note that, as the CCD used here only works in the visible band, the infrared components of diffraction cannot be seen from Fig. 4(c). In addition, in Fig. 4(d), the measured reflection or diffraction efficiencies of zero- and two first-order modes are presented for the wavelength 600 ~ 1000 *nm* (in this region, the first-order mode has a diffraction angle *θ*_01_ = 19.5^o^ ~ 33.7^o^, not overlapping the second-order one where *θ*_02_ = 41.8^o^ ~ 90^o^). Indeed, the zero-order (0, 0) and first-order (0, 1) modes own the smaller diffraction efficiency, especially in the long wavelength region (less than 10%). On the contrary, the first-order (0,−1) mode presents an increased efficiency in the whole measured spectral range (in the wavelength 750 ~ 1000 *nm*, the efficiency is higher than 60%). The experimental results are close to the numerical simulations and some deviations can be attributed to the fabrication errors in the FIB milling.

## Discussions

In conclusion, ultra-broadband and strongly enhanced diffraction of light from a thin and planar metasurface has been suggested. The effect is associated with the wideband and enhanced light scattering of the sandwich unit cells, where localized and self-adaptive oscillations of free electrons can be induced. The subwavelength feature in x direction and super-wavelength feature in y direction enable that only a few propagating diffraction orders can be supported by the system. We found that, for the simple rectangle metal patches, zero-order reflection can be strongly suppressed, leading to two symmetrical, wideband, and enhanced first-order diffractions. The degeneracy of the two first-order diffractions can be lifted with the asymmetrical trapezoid metal patches, yielding highly-efficient and ultra-broadband diffraction in only one of the two orders. In addition to the interests in the spectroscopy study and thin-film solar cell designs, the strongly enhanced diffraction could also have some interesting mechanical effects. For example, due to the asymmetrical and enhanced first-order diffraction, an enhanced lateral optical force can be induced on the flat metasurface by the normally incident light.

## Methods

### FDTD simulations

The diffraction spectra and electromagnetic field distributions were simulated with the commercial software package FDTD Solutions 8.6 (Lumerical Solutions, Inc., Canada). To calculate the diffraction spectra, periodic boundary conditions were used in the x and y directions, and open boundary conditions were used in the z direction. To calculate the energy flow distribution, a finite width of structure (19 lattice periods in the y direction) has been employed; the plane wave of finite width was incident on the structure, illuminating 10 lattice periods (in the y direction). In this case, periodic boundary condition was used in the x direction, and open boundary conditions were set in the y and z direction. In addition, to calculate the scattering cross section and near-field patterns of a single unit cell, open boundary conditions were used for all the directions. In the simulations, the refractive index of glass was set as *n*_*d*_ = 1.5, and the permittivity of metal (gold) was modeled by the Drude dispersion 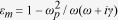, where 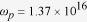 rad/s and 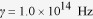.

### Fabrication and measurement

In the experiment, a planar gold-glass-gold trilayer with the thickness 80/90/150 nm were deposited, respectively, by the magnetic sputtering on the glass substrate. The upper trapezoid patches with the designed sizes were then milled in the 80 nm-thick gold film by the focused-ion-beam (FIB) system (Helios 600i, FEI Co., 30 KeV Ga^+^ ions, 7 pA beam current). The whole array consists of 140*30 units with the side length around 50 μm. In the measurement, the x-polarized incident light (with the working wavelength around 500 ~ 1000 nm) was incident normally upon the sample. The discrete zero-order and higher-order diffractions were collected by an optical spectrum analyzer (ANDO AQ-6315 A).

## Author Contributions

Y.Z. and C.P.H. conceived the design. Y.Z. and L.Z. performed the numerical simulations. Q.J.W. fabricated the samples and J.Q.L. performed the measurements. Y.Z. and C.P.H. wrote the paper.

## Additional Information

**How to cite this article**: Zhang, Y. *et al.* Ultra-broadband and strongly enhanced diffraction with metasurfaces. *Sci. Rep.* **5**, 10119; doi: 10.1038/srep10119 (2015).

## Supplementary Material

Supplementary Information
