# Supplementary material for: Ultra-broadband and strongly enhanced diffraction with metasurfaces
Source: Sci Rep. 2015 May 14;5:10119. doi: 10.1038/srep10119 (PMC4431346; doi:10.1038/srep10119)
Supplement: Supplementary Information [file srep10119-s1.pdf]

Supporting Information

Ultra-broadband and strongly enhanced diffraction with metasurfaces

Yong Zhang, Lin Zhou, Jia-qi Li, Qian-jin Wang, and Cheng-ping Huang

Here we analyze how the structure parameters of a single rectangle or trapezoid patch affect the zero- and first-order diffractions of the periodic metasurface. Note that, with the variation of structure sizes, the qualitative conclusion, i.e., the ultra-broadband and strongly enhanced diffraction, can still be obtained in certain wavelength regimes.

I. Diffraction spectrum of rectangle metal sandwiches

We first numerically investigate the effect of length l, width a, and thickness t of the rectangle patch, as well as the thickness h of the glass spacer on the diffractions of the rectangle patch array. The obtained results are shown in Figure S1-S4. First of all, the basic structure parameters of the rectangle patch are chosen as: the length is l=600 nm, the width is a=200 nm, the patch thickness is t=80 nm and the spacer thickness is h=90 nm. The lattice periods in the xy plane were set as d_x=360 nm and d_y=1800 nm. As one parameter changes, the other parameters keep on the above values.

As can be seen from Figure S1 to S4, the length, width, thickness of the rectangle patch and the thickness of the glass spacer all can affect the zero- and first-order diffractions. Especially, the diffractions are most sensitive to the width a and length l of the rectangle patch (see Figure S1 & S2). In contrast, the thickness t and h have relatively weak impact on the diffractions (see Figure S3 & S4). With the variation of a, the working wavelength range and the bandwidth corresponding to the high efficiency first-order diffraction is changed accordingly. The reason is that the free electrons in the sandwiches oscillate along the width of the metal patches and the ground metal plane. The larger the width of metal patch, the longer the oscillating

period and the larger the allowed working wavelength. When *a*=150 nm, the first-order diffraction is dominant in the short-wavelength regime. When *a*=200 nm, the first-order diffraction in the long wavelength is improved obviously. When *a*=250 nm, the first-order diffraction is almost flat in the whole wavelength band.

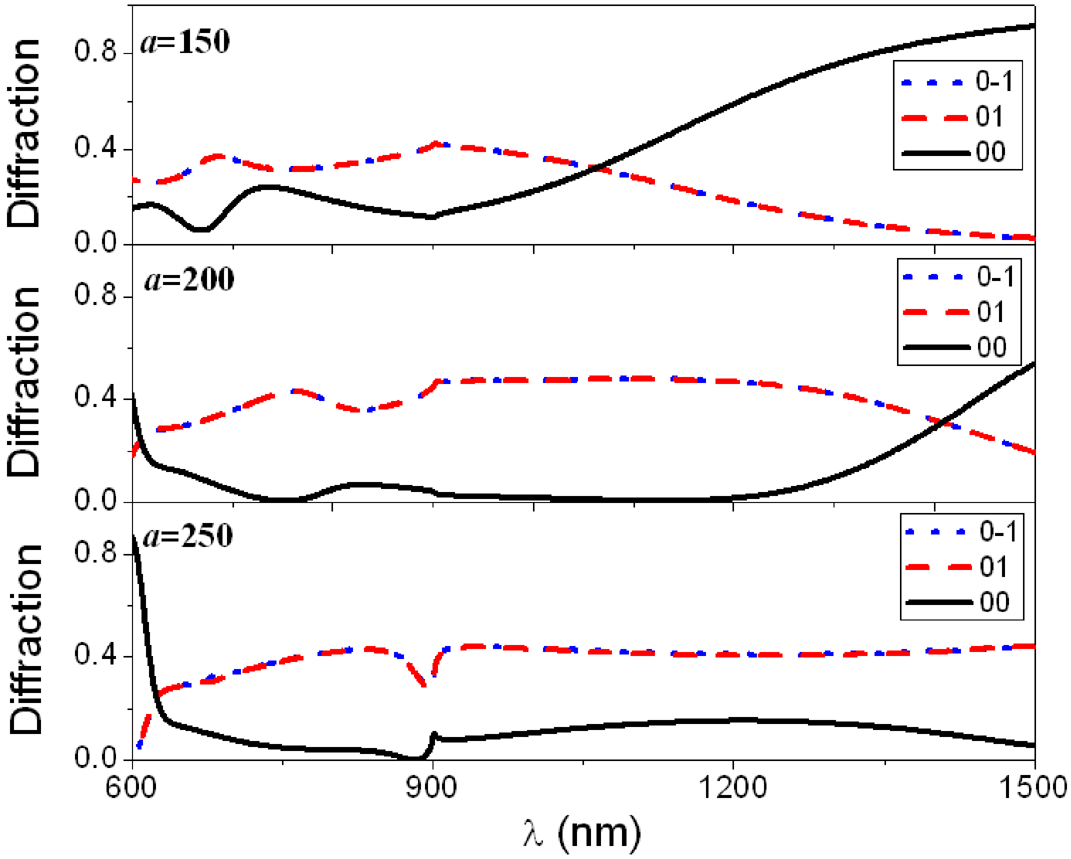

Figure S1. The effect of width *a* of the rectangle patch on the diffraction.

The diffractions are also sensitive to the length *l* of the rectangle patch. In the paper, we have chosen *l*=600 nm. From Figure S2, we can see that, when *l* is decreased (*l*=400 nm), the first-order diffraction will be degenerated and zero-order diffraction enhanced instead. However, when *l* is further increased (*l*=1000 or 1400 nm; especially when *l* is close to the lattice period d_y), the zero-order mode is also boosted, suppressing the first-order diffractions.

We think there are three channels for the reflection of incident light. The first channel is the upper surface of the metal patches, which can reflect part of incident

light directly. When the area of metal patch is close to that of unit cell, this reflection channel will be dominant. The second channel is the ground metal plane, which can also reflect part of incident light directly. When the area of metal patch is significantly decreased, this reflection channel will be dominant instead. The third channel is the metal sandwiches discussed in the paper, which can grasp part of incident light and present enhanced light scattering. There is a competition among the three channels. To maximize the coupling of light to the metal sandwiches, there should be an optimal length of metal patches, as the much larger or much smaller metal patches are beneficial to the first and second channels. Consequently, when the length *l* is small or large enough (Figure S2), the zero-order reflection due to the first and second channels will be enhanced, leading to degeneration of the first-order diffraction.

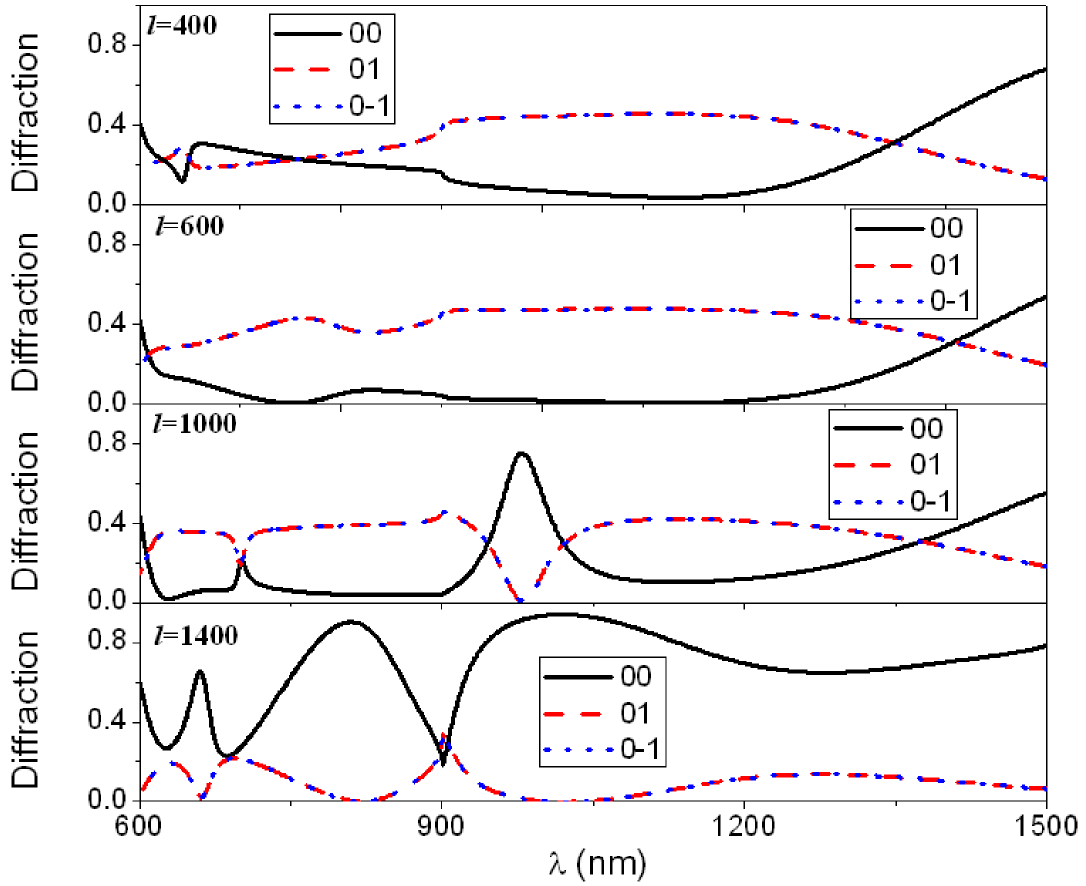

Figure S2. The effect of length *l* of the rectangle patch on the diffraction.

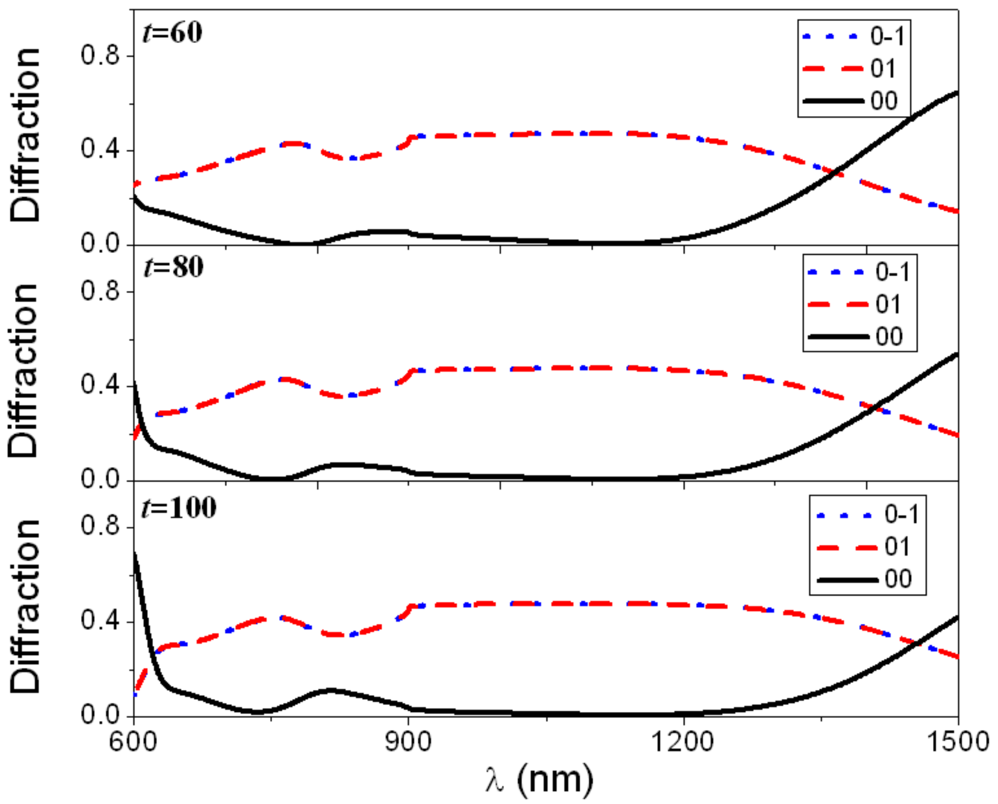

Figure S3. The effect of thickness *t* of the rectangle patch on the diffraction.

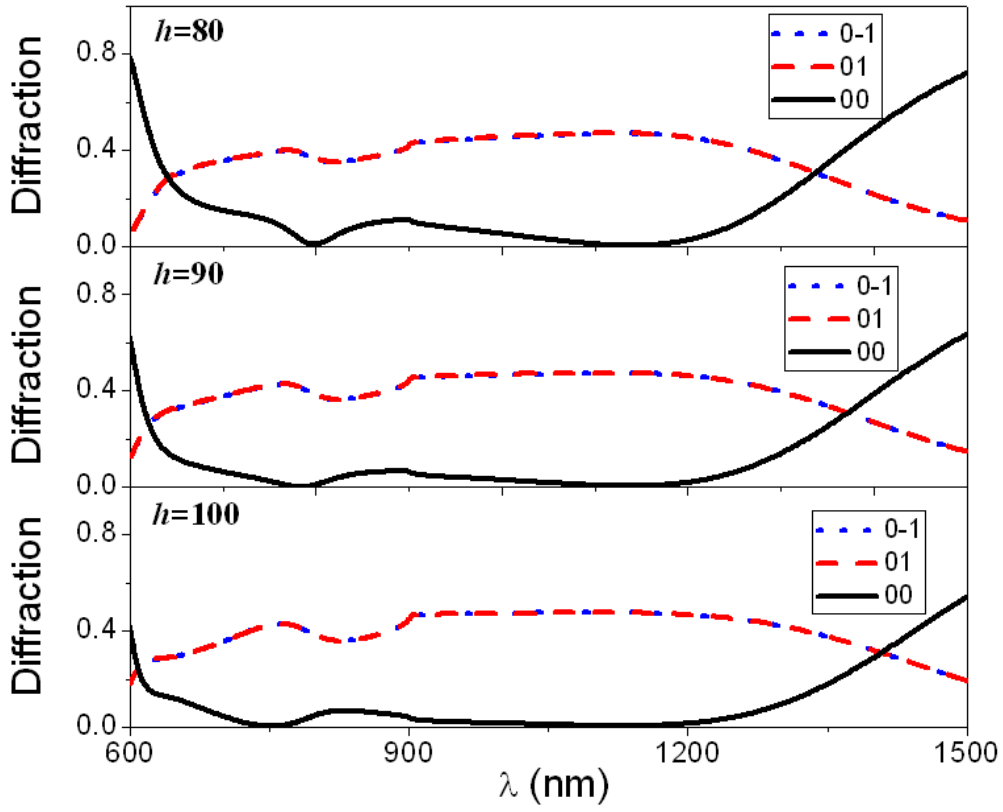

Figure S4. The effect of thickness of the glass spacer *h* on the diffraction.

II. Diffraction spectrum of trapezoid metal sandwiches

The influence of the length of the upper and lower trapezoid bases *a* and *c* and the height of the trapezoid *b* on the zero- and first-order diffractions of the trapezoid patch array are also explored numerically. The obtained results are shown in Figures S5-S7. The basic structure parameters of the trapezoid patch are chosen as *a*=100 nm, *b*=1200 nm, and *c*=320 nm; the thickness of the trapezoid patch and the glass spacer are set as *t*=80 nm and *h*=90 nm, respectively; the lattice periods in the *xy* plane were set as d_x=360 nm and d_y=1800 nm, respectively. As one parameter changes, the other parameters keep on the above values. The effect of thickness of the trapezoid patch and the glass spacer on the diffractions is similar to that of the rectangle patch array and the results will not be given here.

From Figure S5-S7, we can see that all the three parameters *a*, *b*, and *c* have impact on the zero- and first-order diffractions. For the base *a*, we change its value from 100 to 200 and 300 nm, corresponding to the evolution from an asymmetrical to almost symmetrical structure. A change of the two first-order diffractions from splitting to near degeneration can be seen clearly. Moreover, the increase of base length *a* can increase the area of trapezoid metal patches, thus enhancing the zero-order mode related to the first reflection channel mentioned above (and thus reducing the first-order diffractions).

Figure S7 shows the influence of the height *b* of the trapezoid on the diffractions. The results are somewhat similar to that of rectangle patch arrays. When *b*=1200 nm, an enhanced and ultra-boradband first-order diffraction can be obtained. When *b* is decreased to 800 nm, the second reflection channel related to the ground metal plane is enhanced, reducing the first-order mode in the long-wavelength region. On the contrary, when *b* is further increased, the first reflection channel related to the gradient trapezoid-patch surface will be enhanced, thus reducing the first-order mode as well. The latter becomes extremely obvious when *b*=1800 nm (that is to say, the trapezoids connect each other in the y direction, forming a grating-like structure). In this case, the enhanced and asymmetrical first-order diffraction only remains in the long-wavelength band. Thus, optimal patch sizes are required for the practice.

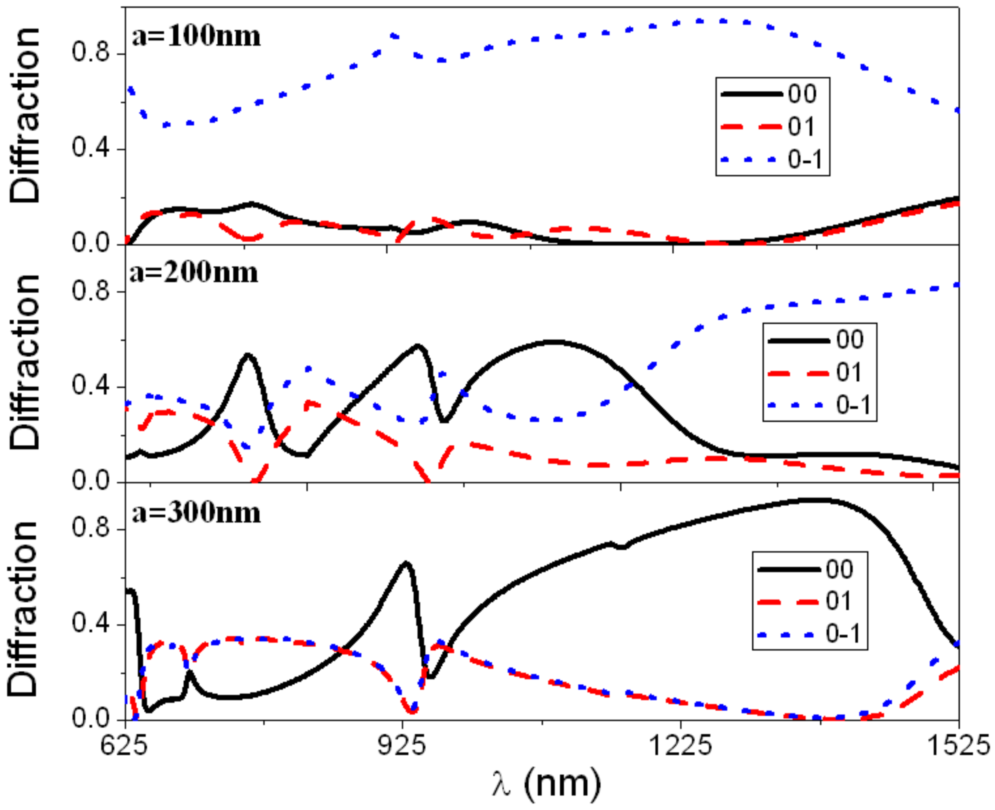

Figure S5. The effect of length of the upper trapezoid edge *a* on the diffraction.

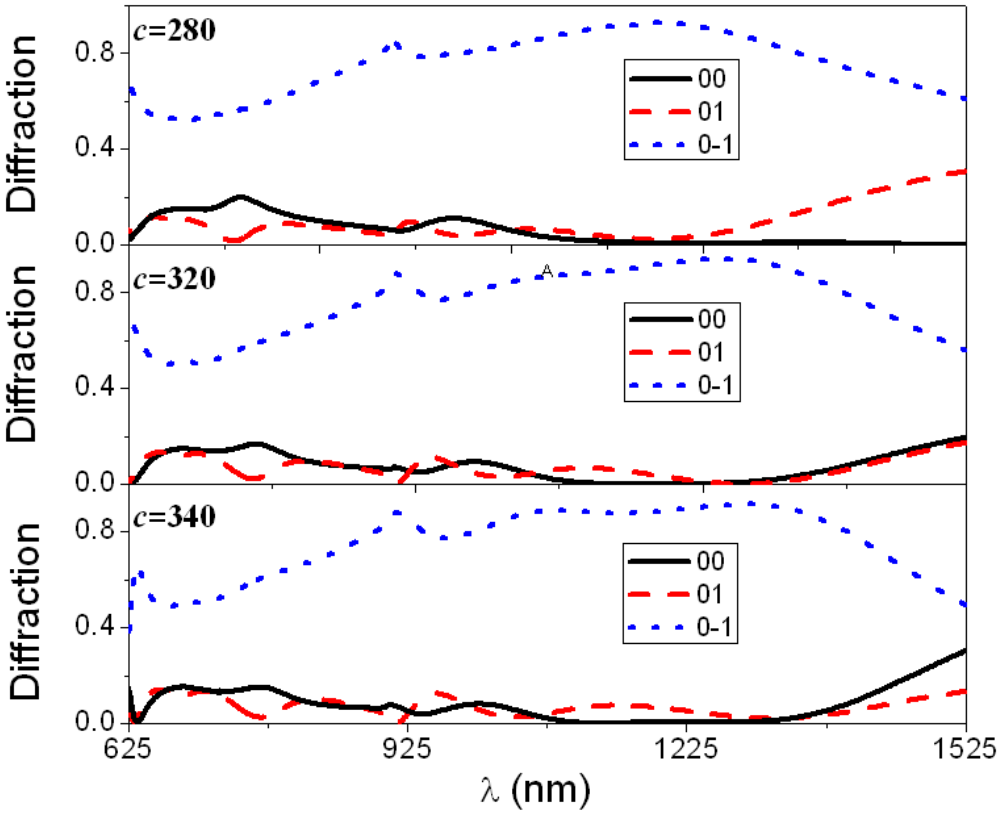

Figure S6. The effect of length of the lower trapezoid edge *c* on the diffraction.

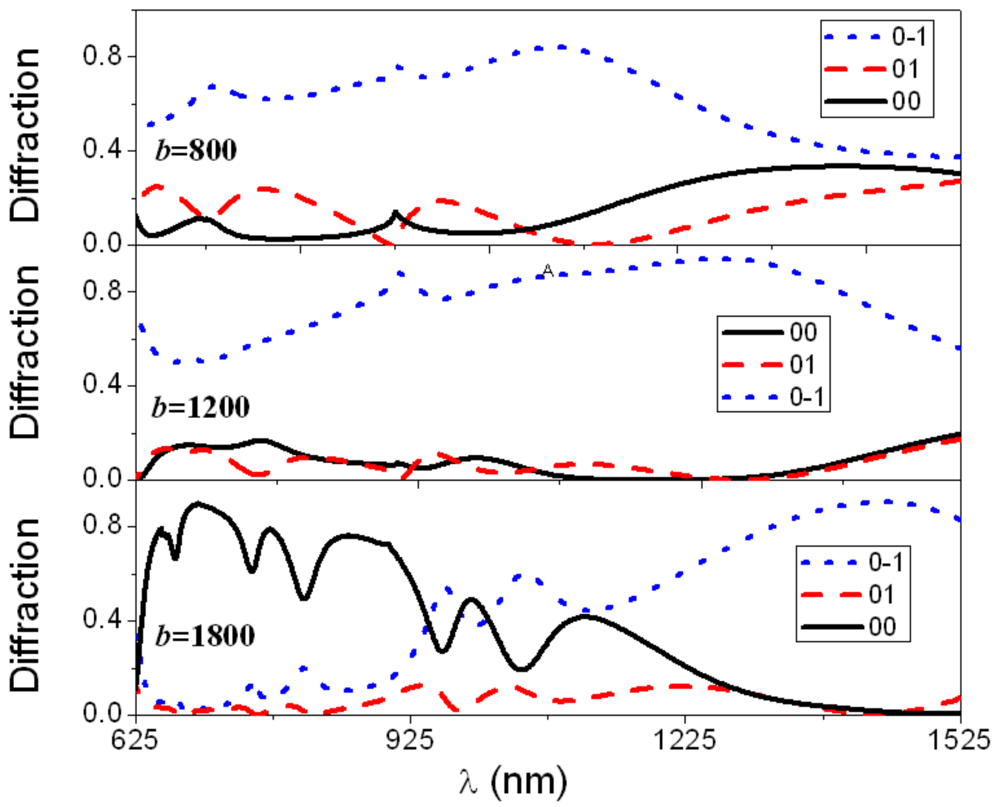

Figure S7. The effect of the height of the trapezoid *b* on the diffraction.
